# Among Montreal Graduates

**Published:** 1898-07

**Authors:** 


### Among Montreal Graduates.

Dr. Alexander Glass, of the class of 1882, has become wedded to a naphtha launch, and the streams adjacent to Philadelphia find daily the bow of the "Corona" plying into all their nooks and coves.

Dr. M. C. Baker, of the class of 1879, has found no spot so attractive as the city of his alma mater, and no place so good to be as within the old college walls.

Dr. A. W. Clement, of the class of 1883, was known in college as the wizard of the microscope, and for many years after followed closely its teachings in national and State work.

Dr. N. P. Hinkley, of the class of 1880, was as popular as a student as he was an open debater and defender of his convictions in college days at Montreal.

Dr. William Jakeman and the late Dr. J. M. Skally, of the class of 1880, were boon friends in their college days, and Jakeman was known as father of the boys.

It is said of Dr. Benjamin D. Pierce, of the class of 1881, that in those days America did not possess a trotting-bred horse whose pedigree and record were not at his tongue's end. He put many to sleep in his college days when the point of six removes from the sire and dam was reached.

Dr. C. B. Robinson, of the class of 1882, was known as among the most emphatic in expression, and clinched his arguments with strong words. He early demonstrated his faculties for a successful career in the profession.

Dr. A. H. Baker, of the class of 1876, won its medal, and thus pointed the way for a career in rearing another college, where to others might be imparted a portion of the knowledge obtained at Montreal.

Dr. Richard Price, of the class of 1883, or Dick, as the boys all called him, was full of prospects and a noted castle-builder, and lived to touch fame and fortune in the great Northwest in the better days of the profession.

Dr. W. L. Williams, of the class of 1879, was as steady a worker in college days as he has proven a roving veterinarian in the past six to eight years, claiming a residence in more divergent points of the compass than any of his classmates.

Dr. Paul Paquin, of the class of 1883, early demonstrated his fitness for a career of investigation, and has ridden his hobby ever since, winning fame and wooing fortune.

Dr. A. R. Rowat, of the class of 1887, found more congenial the climate of the Sandwich Islands, and now revels in the feeling of again being a connection of North America.

Dr. V. T. Daubigny, of the class of 1879, continued his successful studies after leaving college by connecting himself with the Veterinary Department (French School) of Laval University.

Dr. Frank H. Miller, of the class of 1887, early demonstrated a fondness for canine pathology, and follows now exclusively the bend of his mind in early years.

Dr. D. Lemay, of the class of 1879, was the pride of his classmates, and his early seeking of military associations the natural trend of all his early promises.

Dr. J. M. Parker, of the class of 1889, was a jolly good fellow at college, red-headed and hopeful at all times, and the " hole in the bottom of the sea " never ruffled his brain.

Dr. John Ryan, of the class of 1877, found the northern climate of Canada too slow, and only the rush and bustle of the " Windy City " of the West proved a fitting place for his labors.

Dr. Cecil French, of the class of 1894, won the medal for that year, and his fondness for the more domesticated animals found only its full sway in the " City of Magnificent Distances," where canine and feline pets abide in every household.

Dr. C. C. Lyford, of the class of 1879, was noted as a good fellow, and has ever maintained the kindest and warmest regard of every one among the profession whose friendship he formed.

Dr. James B. Paige, of the class of 1888, was a faithful plodder after knowledge, and this drift of mind has led him to the fields of instruction at home and abroad.

Dr. Joseph Plaskett, of the class of 1892, or " Joe," as he was wont to be called, was a Southern representative who maintained well the fair name and worth of a Southern gentleman.

Dr. J. Robertson, of the class of 1888, longed for a military career, and found his much-hoped-for experience in the present Hispano-American war, but recent advices announce him among the wounded.

Dr. L. E. Willyoung, of the class of 1890, was deservedly popular among his classmates, and the " Queen City of the Lakes " welcomed him to a popular place among her veterinarians.

Dr. Charles McEachran, of the class of 1884, was a true advocate of the value of a sporting turn of mind, and many happy hours following the hounds over hill and heather have been spent since his college days.

Drs. Benjamin D. Pierce, of Springfield, Thomas Blackwood, of Boston, and J. R. McLaughlin are a trio of the older graduates of Montreal in the "Bay State."

Drs. John M. Parker, of Haverhill, Mass.; Frank H. Miller, of New York, and A. W. Clement, of Baltimore, are examiners at the closing examinations at McGill. These three represent the United States on the Board, and are nominated by the Provincial Government for these duties.

Drs. J. A. Couture, of Quebec; A. McCormic, of Ormstown, and A. W. Harris, of Ottawa, represent the Canadian graduates on the Board of Examiners.

Prof. Wesley Mills, of the class of 1890, still lingers abroad on leave of absence, enjoying himself among the storehouses of knowledge in Germany. He will resume his teachings at McGill in October.

Dr. A. E. Moore, of the class of 1894, stands as representative fellow in comparative medicine and veterinary science in the corporation of McGill University.

